# The Study on the Agreement between Automatic Tongue Diagnosis System and Traditional Chinese Medicine Practitioners

**DOI:** 10.1155/2012/505063

**Published:** 2012-08-08

**Authors:** Lun-chien Lo, Yung-Fu Chen, Wen-Jiuan Chen, Tsung-Lin Cheng, John Y. Chiang

**Affiliations:** ^1^Department of Traditional Chinese Medicine, Changhua Christian Hospital, Changhua 500, Taiwan; ^2^Graduate Institute of Statistics and Information Science, National Changhua University of Education, Changhua 500, Taiwan; ^3^Department of Healthcare Administration, Central Taiwan University of Science and Technology, Taichung 402, Taiwan; ^4^Department of Health Services Administration, China Medical University, Taichung 402, Taiwan; ^5^Department of Computer Science & Engineering, National Sun Yat-sen University, Kaohsiung 424, Taiwan

## Abstract

Tongue diagnosis is an important practice in traditional Chinese medicine (TCM) for diagnosing diseases before determining proper means of treatments. Traditionally, it depends solely on personal knowledge and experience of the practitioner, thereby being criticized as lacking of objectivity. Currently, no research regarding intra- and inter-agreements of automatic tongue diagnosis system (ATDS) and TCM doctors has been conducted. In this study, the ATDS is developed to extract a variety of tongue features and provide practitioners with objective information to assist diagnoses. To evaluate the ATDS clinical stability, 2 sets of tongue images taken 1 hour apart from 20 patients with possible variations in lighting and extruding tongue, are employed to investigate intra-agreement of the ATDS, intra-agreement of the TCM doctors, and the inter-agreement between the ATDS and TCM doctors. The ATDS is shown to be more consistent with significantly higher intra-agreement than the TCM doctors (kappa value: 0.93 ± 0.06 versus 0.64 ± 0.13) with *P* < 0.001 (Student's *t*-test). Inter-agreements between the ATDS and TCM doctors, as well as among the TCM doctors are both moderate. The high agreement of the ATDS can provide objective and reliable tongue features to facilitate doctor in making effective observation and diagnosis of specific diseases.

## 1. Introduction

**Figure 1:**
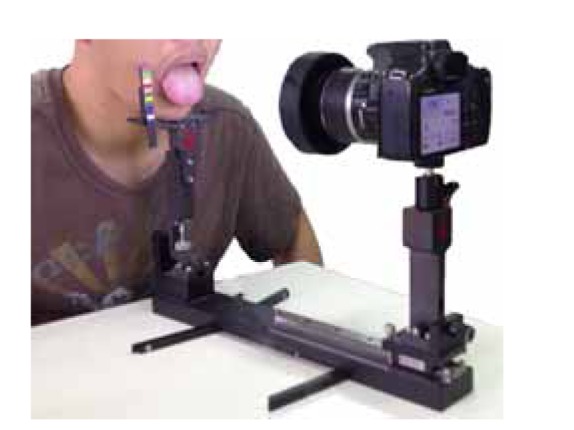
Illustration of tongue image capturing with the ATDS.

In recent years, traditional Chinese medicine (TCM) has received wider acceptance and warmer embrace from western medicine. Several previous studies have been conucted on the issue of consistency of TCM [1–11], and the results have indicated that inter- and intra-observer agreements are low. However, those studies only focused on intra-observer agreement among practitioners. From the best of our knowledge, no research with regard to intra- and inter-observer agreements of the automatic tongue diagnosis system (ATDS) and TCM practitioners has been conducted so far. In light of this observation, in this study, the ATDS was developed to extract tongue features to assist the diagnosis of TCM practitioners, as shown in Figure 1 [12]. This paper investigates the levels of intra-observer agreement in tongue diagnosis for both ATDS and TCM doctors, and the inter-observer agreement between ATDS and TCM doctors.

Tongue diagnosis plays an important role in TCM [13]. The tongue is connected to the internal organs through meridians; thus the conditions of organs, qi, blood, and body fluids as well as the degree and progression of disease are all reflected on the tongue [14, 15]. Organ conditions, properties, and variations of pathogens can be revealed through observation of tongue. For example, changes in the tongue proper primarily reflect organ status and the flow of qi and blood; variations in tongue fur can be employed to determine the impact of exogenous pathogenic factors and the flow of stomach qi. In clinical practice, practitioners observe tongue characteristics, such as the color and shape, and the amount of saliva before deducing the primary ailment of a patient. However, observation diagnosis is often biased by subjective judgment, originating from personal knowledge, experience, thinking patterns, diagnostic skills, and color perception or interpretation. There are no precise or existing quantifiable standards. Different practitioners may pass varying judgments on the same tongue, while a practitioner may even reach different diagnoses on the identical tongue if examined at different time. It was reported that the mean intrapractitioner agreement reached 61%, while the interpractitioner agreement was as low as 18.2% between 30 practitioners observing 10 tongue images [11]. Such inconsistency causes many people to be skeptical of TCM, which raised our motivation to develop the ATDS [16]. It is expected that ATDS can assist TCM practitioners to establish reliable diagnoses by providing them with standardized procedures as well as objective, reliable, and quantified data.

To be employed in clinical inspections, the reliability of ATDS in the face of environmental lighting variations and differences, for example, orientation, forces applied in extruding tongue, and so forth, presented in tongue images taken at different time, must be studied first in order to gain the trust of practitioners and patients. The aims of this study are (a) to compare the intra-observer agreement of the ATDS and TCM doctors assessments, respectively, and (b) to evaluate the inter-observer agreement between the ATDS and TCM doctors assessments of tongue features.

## 2. Materials and Methods

### 2.1. Participants

A group of doctors, consisting of 12 experienced TCM practitioners having clinical experience ranging from 3 to 15 years with a mean of 5.5 years from the Chinese medicine department at Changhua Christian Hospital (CCH) in Taiwan, were asked to participate in this study. All of them were trained in Taiwan, each holding a BS degree. Some of them own MS or Ph.D. degrees. They were asked to attend regular weekly meetings in the past two years to examine over 1000 tongue images collected through the outpatients of CCH. The images with the consensuses reached by this group of doctors were employed as the training data for developing the ATDS [12]. The same group of 12 TCM doctors, who reached consensus on the 1000 tongue images, were further invited to participate in the agreement tests described later.

### 2.2. Automatic Tongue Diagnosis System

**Figure 2:**
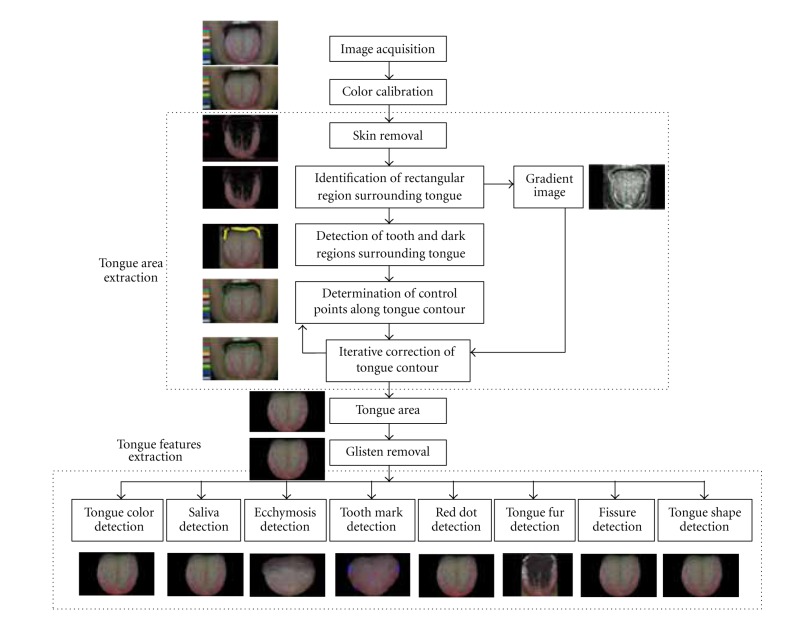
The processing flow of ATDS analysis.

As shown in Figure 2, ATDS includes two major portions: image capturing and feature analysis [16]. The consistency and stability of image capturing relies on brightness and color calibration to compensate for variation such as intensity and color temperature of light source and imaging hardware, for example, the type of cameras or their settings including resolution, shutter speed, aperture, and white balance. Analysis of tongue images proceeds in two steps, namely, isolation of the tongue region within an image and extraction of tongue features. The purpose of isolating the tongue region is to eliminate irrelevant lower facial portions and background surrounding the tongue, thereby facilitating feature identification and extraction. The extraction of tongue features employs criteria such as the aspect ratio, color composition, location, shape, and color distribution of the tongue, as well as the quantity of neighboring pixels. Distinguishing characteristics employed in tongue diagnosis are extracted, such as tongue color, tongue fissure, fur color, fur thickness, ecchymosis, tooth mark, red dot, saliva, and tongue shape, to further generate detailed information regarding length, area, moisture, and number of fissures, marks, and dots. Based on the suggestions of TCM doctors participating in this investigation, ATDS selects 9 primary tongue features, including tongue color (slightly white, slightly red, red, dark red, and dark purple), fur color (white, yellow, and dye), fur thickness (none, thin, and thick), saliva (none, little, normal, and excessive), tongue shape (thin and small, moderate, fat, and large), tongue fissure, red dot, ecchymosis, and tooth marks (the last four are divided into categories of none, mild, moderate, and severe), for intra- and inter-agreement studies.

### 2.3. Questionnaire

**Figure 3:**
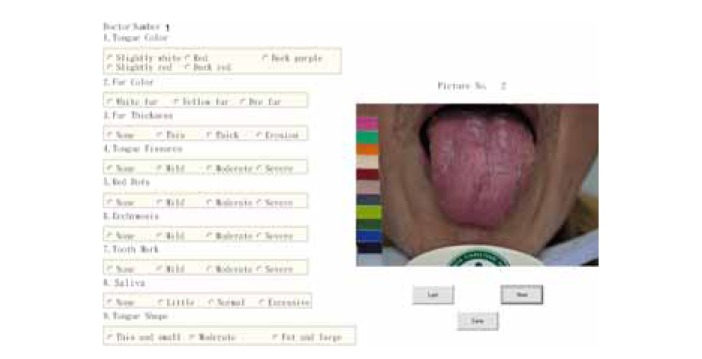
A snapshot of the computerized tongue diagnosis questionnaire.

Through discussion with the tongue diagnosis team at CCH, nine primary features, namely, tongue color, fur color, fur thickness, saliva, tongue shape, tongue fissure, red dot, ecchymosis, and tooth mark were included in the tongue diagnosis questionnaire, as shown in Figure 3. The tongue features identified and the classification of each feature are more diverse than previous studies [11, 13–15].

The questionnaires were conducted automatically by a computer program. The display screen was color calibrated using Datacolor Spyder 3 ELITE before experiment. The same set of tongue images shown to each of the 12 doctors were displayed in a random order.

### 2.4. Experiment Procedure

#### 2.4.1. Image Samples

**Figure 4:**
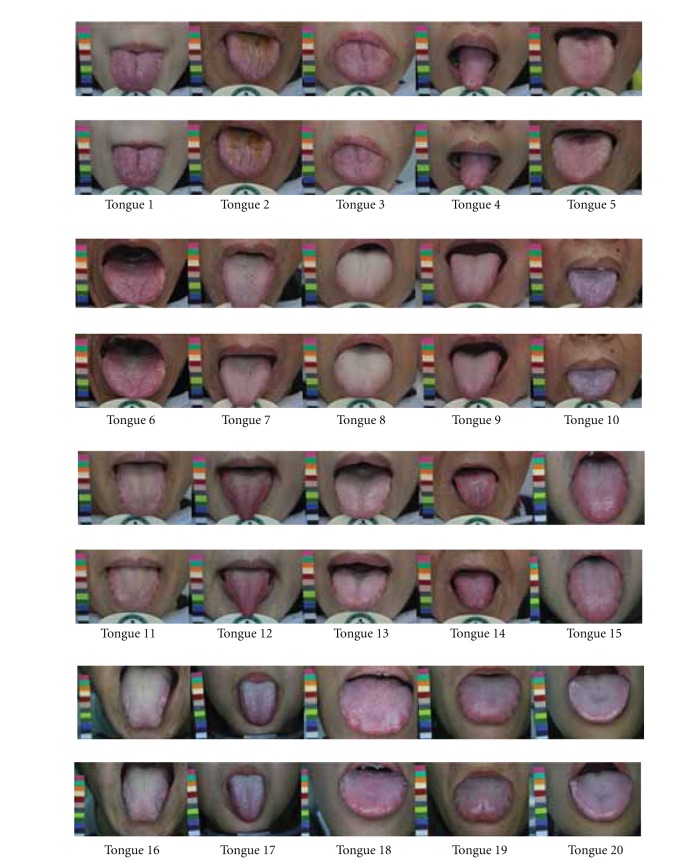
Samples of 20 matching pairs of tongue images, the upper and lower tiers of images are taken from the same patients separately at a one-hour interval.

As shown in Figure 4, two sets of tongue images taken 1 hour apart from 20 patients were employed as samples for conducting the experiments. At time *T*_1_, the first set of tongue images *P*_*T*__1_ = {*P*_*T*__1_(*i*) | *i* = 1,2,…20} of 20 patients were taken using ATDS first. Next, at time *T*_2_, after an interval of one hour, the second set of tongue images *P*_*T*__2_ = {*P*_*T*__2_(*i*) | *i* = 1,2,…20} were captured again from the same group of patients. The matching image pairs *P* = {*P*_*T*__1_(*i*), *P*_*T*__2_(*i*) | *i* = 1,2,…20} of the tongue images, taken separately at a one-hour interval for 20 subjects, were employed as the study samples. 

#### 2.4.2. Agreement Test

**Intraobserver Agreement**

The intra-observer agreements were conducted for both the ATDS and the group of 12 TCM doctors. The 20 matching pairs of tongue images pair *P* were analyzed by the ATDS to derive the intra-observer agreement of the ATDS through the statistical analysis of the features extracted from the tongue images. On the other hand, each of the participating TCM doctors observed the images *P*_*T*__1_ and filled out the questionnaire. After a week, the TCM doctors were asked to review the same set of images *P*_*T*__1_, presented with a different order, and filled out the questionnaire again. The intra-observer agreement of the TCM doctors was evaluated according to the answers collected in the two-stage questionnaires.

**Interobserver Agreement**

According to the diagnostic results of the image set *P*_*T*__1_ determined by the ATDS and TCM doctors, respectively, the strength of inter-agreement between the ATDS and TCM doctors assessments of tongue features was evaluated.

### 2.5. Data Analysis

For tongue and fur color, the intra-agreement and inter-agreement of ATDS and DC were represented by Cohen's kappa coefficient [17]. Cohen's kappa treats all disagreements equally. The weighted kappa incorporates the magnitude of each disagreement and provides partial credits for disagreements when complete agreement is not reached. In fur thickness, tongue fissure, red dot, ecchymosis, tooth mark, saliva, and tongue shape, agreement is expressed by a quadratic-weighted kappa coefficient [18]. The inter-agreement among TCM doctors is indicated with Fleiss' kappa coefficient [19], which is a statistical measure for assessing the reliability of agreement between a fixed number of raters when assigning categorical ratings to a number of items or classifying items. While Cohen's kappa is only applicable to assessing the agreement between two raters, the measure of agreement proposed by Landis and Koch [20], on the other hand, is divided into six levels according to the kappa value: poor (less than 0.00), slight (0.00 to 0.20), fair (0.21 to 0.40), moderate (0.41 to 0.60), substantial (0.61 to 0.80), and almost perfect (0.81 to 1.00).

In this study, the level of intra-observer agreements for the ADTS and the TCM doctors was measured using Cohen's kappa coefficients. The matching image pairs of tongue images were taken 1 hour apart from 20 patients. The intra-observer agreements of the 12 TCM doctors were calculated based on the same image set *P*_*T*__1_ observed at two different periods a week apart, and then the mean values and standard deviations (STDs) of individual tongue features were compared with ADTS. Inter-observer agreements between ATDS and the group of TCM doctors and among TCM doctors were measured using Fleiss' kappa coefficients. 

Since the intra-observer agreement of the ATDS was analyzed with Cohen kappa according to the 2 set of 20 matching pairs of tongue image *P*, a single kappa value was obtained for an individual tongue feature. On the other hand, 12 kappa values were obtained from 12 TCM doctors with regard to each of the tongue features, which were then averaged to be compared with the ATDS. Therefore, one-sample *t*-test was applied to test the intra-observer agreement between the ATDS and the group of TCM doctors regarding the 9 individual tongue features with the kappa value of each tongue feature assigned as the hypothetical mean value. Furthermore, the means and STDs of the 9 tongue features were compared with two sample *t*-test. The significant level is set as *P < *0.05.

## 3. Results

Table 1 shows an example of tongue feature, that is, tongue color, evaluated by 12 TCM doctors for 20 patients. As shown in Figure 3, the nominal values indicate different characteristics of tongue color with 1, 2, 3, 4, and 5 representing slightly white, red, dark purple, slightly red, and dark red, respectively. As shown in this table, the observations of tongue color for patients 5, 8, 10, 11, 12, and 16 by 12 TCM doctors are very consistent with 100% consensus, while patients 2, 9, and 19 demonstrate greater difference in opinion.

### 3.1. Intra-Observer Agreement

The results of intra-observer agreement analysis for tongue characteristics between the ATDS and TCM doctors are listed in Table 2. For ATDS, the kappa value (range: 0.84–1.0, mean: 0.93 ± 0.06) is significantly better (two sample *t*-test,* P < *0.001) than that of TCM doctors (range: 0.44–0.83, mean: 0.64 ± 0.13) by considering the mean of 9 tongue features. Regarding individual tongue features, except the tongue shape (one sample *t*-test, *P > *0.05), the kappa values of all the other 8 tongue features of the ATDS are significantly higher than the TCM doctors. 

### 3.2. Inter-Observer Agreement

The results of the inter-observer agreement analysis between ATDS and group of TCM doctors are tabulated in Table 3. The kappa values range from 0.25 to 0.79 with a mean of 0.45 ± 0.17, indicating a moderate inter-observer agreement.

The results of the inter-observer agreement analysis among TCM doctors are listed in Table 4. The kappa values range from 0.16 to 0.62 with a mean of 0.41 ± 0.15, representing a moderate inter-agreement.

## 4. Discussion and Conclusion

The purpose of measuring the intra-observer agreement in ATDS is to test the system's reliability under various lighting, image-taking angles, and tongue lengths. In other words, the system is tested as to whether it could derive the same conclusions in different shooting conditions. The results reveal a mean kappa value of 0.93 (almost perfect), indicating that external factors have only slight impact on the performance for ATDS. The system demonstrates superiority over a team of TCM practitioners with consensus, whose intra-observer agreement shows a mean of 0.64 (substantial).

**Figure 5:**
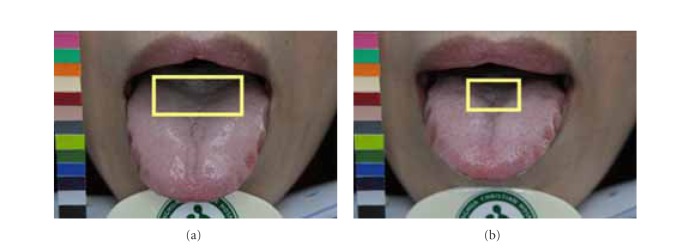
Classifications of fur color and fissure as well as judgment of tooth mark are affected by the length of extruding tongue for the same patient. (a) Long extruding and (b) short extruding tongue.

**Figure 6:**
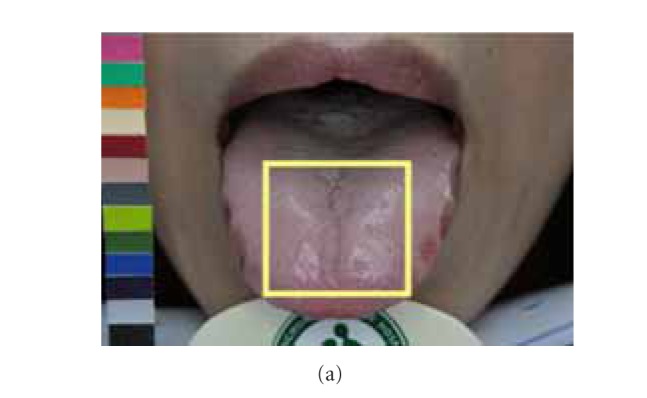
Classifications of saliva and red dots are affected by the image-taking angle between the tongue and camera.

**Figure 7:**
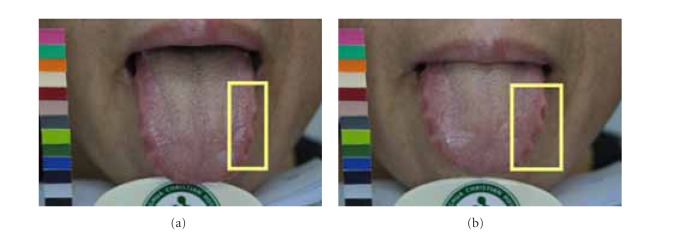
Judgment of tooth mark is affected by different image-taking angle and the length of extruding tongue.

**Figure 8:**
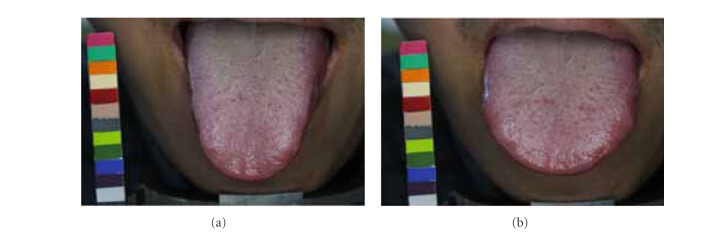
Tongue shape is adversely influenced by the extruding force applied on the tongue.

Due to possible variations in lighting, positioning of chin, and length, shape, and angle of extruding tongue, images taken one-hour apart might exhibit different characteristics. The disparities between matching image pair *P* might lead ATDS to obtain different tongue feature classification. By taking Figure 5 as an example, the tongue extruded on the image in Figure 5(b) is shorter than that in Figure 5(a). The ratio of furs in yellow box to the overall tongue furs is therefore different even for the same patient. Also, longer extruding tongue will expose more fissure areas near the end of tongue, leading to discrepancy in terms of classification of tongue fissure. The angle between the tongue and camera during image capturing will affect the judgment of saliva and the features obscured underneath. In Figure 6, the amount of saliva in the tongue shown in Figure 6(a) is therefore considered to be more than the counterpart in Figure 6(b). The presence of saliva also affects the extraction of red dots underneath. As shown in Figure 7, the judgment of tooth mark is affected by different image-taking angle and length of extruding tongue. Furthermore, as shown in Figure 8, the extruding force applied on the tongue will adversely influence the classification of tongue shape. The inconsistency in image-capturing procedure can be improved through the guidance of a trained technician by following a standard operating procedure (SOP).

**Figure 9:**
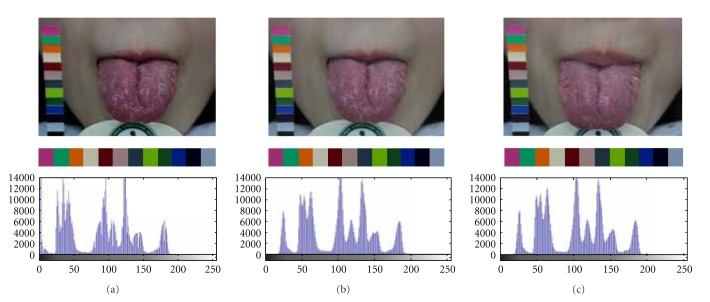
Calibration of image color using the color bar accompanied with the ATDS to make image quality consistent for images taken at difference circumstances. Note that the *x*-axis and *y*-axis of the plots shown on the third row denote gray level and frequency, respectively.

Variations in background lighting may change the color and brightness of the acquired images. One of the significant advantages of the developed ATDS is that it can automatically correct lighting and color deviation caused by the change of background lighting with a color bar accompanied with the ATDS. The color bar placed beside the patient is used for color calibration to make sure that the image quality is consistent even taken at different circumstances. Figures 9(a) and 9(b) display the images taken at *T*_1_ before and after color calibration, respectively, whereas Figure 9(c) demonstrates the image taken at *T*_2_ after calibration. The second and third rows show the color bars clipped from the tongue images and their corresponding histograms. It can be found that the histograms before and after color calibration are quite different with different mean gray scales (86.93 versus 103.68). In contrast, the histograms of the images taken 1 hour apart after color calibration are almost the same with similar mean gray scales (103.68 versus 104.39). 

To prove the feasibility of ATDS in clinical practice, the unavoidable challenge is to determine whether judgment reached by practitioners according to tongue images is consistent with that from ATDS. To this end, this study investigated the inter-observer agreement between characteristic information captured and analyzed by the ATDS and through visual observation by the TCM doctors. The results derived present a mean kappa value of 0.45 (moderate). Even through regular discussions, different TCM practitioners only reach moderate degree (0.41) of inter-observer agreement. This indicates that observation diagnosis is highly influenced by the subjectivity of judgment originating from personal knowledge, experience, thinking patterns, diagnostic skills, and color perception or interpretation. This is especially noticeable for red dots (0.23, fair) and ecchymoses (0.16, fair). The moderate inter-observer agreement obtained is mainly attributed to the divergent judgment among TCM doctors. 

Similar finding was also reported recently by Ko et al. [21]. They investigated inter-observer agreement of tongue observation about stroke patients between two TCM experts with the kappa values ranging from 0.29 to 0.69. Even only two experts were invited to observe the same cases, great variation of inter-observer agreement was still found. This finding is very similar to the results of this study (Table 4, kappa value: 0.16–0.62). In contrast, the intra-observer agreement of ATDS (kappa value: 0.84–1.0, mean: 0.93 ± 0.06) is significantly better than the group of TCM doctors (mean kappa value: 0.39–0.83, mean: 0.64 ± 0.13) with a significant level of *P < *0.001 (two-sample *t*-test). Furthermore, moderate inter-observer agreement between the ATDS and the group of TCM doctors is manifested with a mean kappa value of 0.45 ± 0.17 (Table 3). 

The limitation of this study is that, currently, there is no gold standard to compare the diagnostic accuracy of the tongue images between ATDS and TCM doctors. Therefore, even if the TCM doctors were well trained, poor intra-observer agreement is still manifested with a mean Cohen kappa value of 0.64. The contribution of the ATDS is its great consistency regarding the observation of tongue images, as manifested with great mean kappa value. The intra-observer agreement of the ATDS (0.93 ± 0.063) is significantly better than the TCM doctors (0.64 ± 0.13) with a significant level of *P < *0.001. In conclusion, ATDS is more effective in preventing influence of external factors and can provide TCM practitioners with more objective and precise diagnostic features.

## Figures and Tables

**Table 1:** An example of tongue features, that is, tongue color, evaluated by 12 TCM doctors for 20 patients.

	Doctor											
Patient	D1	D2	D3	D4	D5	D6	D7	D8	D9	D10	D11	D12
P1	4	3	3	3	4	3	4	3	3	4	3	3
P2	5	1	1	5	5	2	5	5	5	2	1	1
P3	2	2	2	2	2	2	2	2	2	2	4	3
P4	5	5	3	5	5	5	5	5	3	5	5	5
P5	2	2	2	2	2	2	2	2	2	2	2	2
P6	4	4	4	4	4	4	5	5	4	4	4	4
P7	3	3	2	3	3	3	3	3	3	3	2	2
P8	2	2	2	2	2	2	2	2	2	2	2	2
P9	5	3	3	4	4	3	5	3	5	4	3	3
P10	2	2	2	2	2	2	2	2	2	2	2	2
P11	2	2	2	2	2	2	2	2	2	2	2	2
P12	2	2	2	2	2	2	2	2	2	2	2	2
P13	2	2	1	2	2	2	2	1	2	2	2	2
P14	2	2	2	3	3	2	2	2	2	2	3	3
P15	3	2	2	2	2	2	2	2	2	2	2	3
P16	2	2	2	2	2	2	2	2	2	2	2	2
P17	3	2	3	3	2	3	2	3	3	3	2	3
P18	2	3	2	2	2	2	2	2	3	2	2	2
P19	3	2	1	1	2	2	1	1	3	2	2	2
P20	2	3	2	2	2	2	2	2	2	2	3	2

**Table 2:** A comparison of intraobserver agreements (kappa values) of the ATDS and TCM doctors.

Features	ATDS	TCM doctors (*N* = 12)
Tongue color^∗∗∗^	1.00	0.39 ± 0.18
Fur color^∗∗^	0.88	0.83 ± 0.04
Fur thickness^∗∗^	1.00	0.68 ± 0.27
Tongue fissure^∗∗^	0.90	0.72 ± 0.18
Red dot^∗∗∗^	0.96	0.50 ± 0.15
Ecchymosis^∗∗∗^	1.00	0.71 ± 0.57
Tooth mark^∗∗∗^	0.94	0.59 ± 0.24
Saliva^∗^	0.86	0.58 ± 0.34
Tongue shape	0.84	0.81 ± 0.09

Mean^†^	0.93 ± 0.06	0.64 ± 0.13

Note: one-sample *t*-test with significance of ^∗^ *P* < 0.05, ^∗∗^ *P* < 0.01, and ^∗∗∗^ *P* < 0.001; two-sample *t*-test with significance of ^†^ *P* < 0.001.

**Table 3:** Interobserver agreement between the ATDS and TCM doctors.

Features	Kappa value
Tongue color	0.43
Fur color	0.40
Fur thickness	0.55
Tongue fissure	0.79
Red dot	0.34
Ecchymosis	0.26
Tooth mark	0.51
Saliva	0.25
Tongue shape	0.52

Mean	0.45 ± 0.17

**Table 4:** Inter-observer agreement among the TCM doctors.

Features	Kappa value
Tongue color	0.50
Fur color	0.62
Fur thickness	0.48
Tongue fissure	0.57
Red dot	0.23
Ecchymosis	0.16
Tooth mark	0.35
Saliva	0.42
Tongue shape	0.40

Mean	0.41 ± 0.15
